# Characterization of STAT3 activation and expression in canine and human osteosarcoma

**DOI:** 10.1186/1471-2407-9-81

**Published:** 2009-03-10

**Authors:** Stacey L Fossey, Albert T Liao, Jennifer K McCleese, Misty D Bear, Jiayuh Lin, Pui-Kai Li, William C Kisseberth, Cheryl A London

**Affiliations:** 1 Department of Veterinary Biosciences, College of Veterinary Medicine, The Ohio State University, Columbus, OH, USA; 2 Department of Veterinary Clinical Sciences, College of Veterinary Medicine, The Ohio State University, Columbus, OH, USA; 3 Center for Childhood Cancer, Nationwide Children's Research Institute and Department of Pediatrics, The Ohio State University, Columbus, OH, USA; 4 Division of Medicinal Chemistry, The Ohio State University, Columbus, OH, USA; 5 Comprehensive Cancer Center, The Ohio State University, Columbus, OH, USA

## Abstract

**Background:**

Dysregulation of signal transducer and activator of transcription 3 (STAT3) has been implicated as a key participant in tumor cell survival, proliferation, and metastasis and is often correlated with a more malignant tumor phenotype. STAT3 phosphorylation has been demonstrated in a subset of human osteosarcoma (OSA) tissues and cell lines. OSA in the canine population is known to exhibit a similar clinical behavior and molecular biology when compared to its human counterpart, and is often used as a model for preclinical testing of novel therapeutics. The purpose of this study was to investigate the potential role of STAT3 in canine and human OSA, and to evaluate the biologic activity of a novel small molecule STAT3 inhibitor.

**Methods:**

To examine STAT3 and Src expression in OSA, we performed Western blotting and RT-PCR. OSA cells were treated with either STAT3 siRNA or small molecule Src (SU6656) or STAT3 (LLL3) inhibitors and cell proliferation (CyQUANT), caspase 3/7 activity (ELISA), apoptosis (Western blotting for PARP cleavage) and/or viability (Wst-1) were determined. Additionally, STAT3 DNA binding after treatment was determined using EMSA. Expression of STAT3 targets after treatment was demonstrated with Western blotting, RT-PCR, or gel zymography.

**Results:**

Our data demonstrate that constitutive activation of STAT3 is present in a subset of canine OSA tumors and human and canine cell lines, but not normal canine osteoblasts. In both canine and human OSA cell lines, downregulation of STAT3 activity through inhibition of upstream Src family kinases using SU6656, inhibition of STAT3 DNA binding and transcriptional activities using LLL3, or modulation of STAT3 expression using siRNA, all resulted in decreased cell proliferation and viability, ultimately inducing caspase-3/7 mediated apoptosis in treated cells. Furthermore, inhibition of either Src or STAT3 activity downregulated the expression of survivin, VEGF, and MMP2, all known transcriptional targets of STAT3.

**Conclusion:**

These data suggest that STAT3 activation contributes to the survival and proliferation of human and canine OSA cells, thereby providing a potentially promising target for therapeutic intervention. Future investigational trials of LLL3 in dogs with spontaneous OSA will help to more accurately define the role of STAT3 in the clinical setting.

## Background

Signal transducers and activators of transcription (STAT) proteins comprise a family of transcription factors that play important roles in cell survival, growth, proliferation, differentiation, apoptosis, metastasis, and angiogenesis [1-3]. Accumulating evidence suggests that constitutively activated STAT3 contributes to tumor development and progression in numerous forms of cancer including those of the breast, head and neck, prostate, skin, ovary, lung, bone, and blood [3-5]. Constitutively activated STAT3 correlates with a more malignant tumor phenotype, resistance to chemotherapeutics, and is associated with decreased survival in some cancers [6-8]. As such, STAT3 may represent a novel target for therapeutic intervention in several cancers. In support of this, a variety of inhibitors of STAT3 have been shown to inhibit tumor cell growth and induce apoptosis both *in vitro *and *in vivo *[1,9,10]. Interestingly, STAT3 is not required for the proliferation of normal cells, and multiple studies have demonstrated that normal cells are more tolerant of loss of STAT3 function. [11].

Constitutive phosphorylation of STAT3 is thought to occur via aberrant upstream signaling, as no naturally occurring activating mutations in the STAT3 gene have been identified [9]. STAT3 is phosphorylated following stimulation of receptor tyrosine kinases by their respective growth factors (i.e, Met/HGF, Kit/SCF), binding of cytokines to their receptors (IL-6, oncostatin M), and by activation of nonreceptor tyrosine kinases such as the Src family kinases (SFKs)[12]. In particular, the SFKs are instrumental in multiple signaling pathways involved in the initiation and/or progression of numerous forms of cancer [13]. Indeed, STAT3 was identified as a phosphorylated substrate of v-src [14] necessary for enabling v-src induced adhesion-independence and malignant transformation [11,12]. STAT3 is now known to be a substrate for SFK members including Fyn and Lyn in addition to Src itself [12,15]. Furthermore, recent studies demonstrated that SFK inhibition in various carcinoma tumor cell lines resulted in loss of STAT3 activity [13].

Although the contribution of STAT3 to epithelial cancers and hematologic malignancies has been described in detail, little is known about the potential role of STAT3 dysregulation in sarcomas. One study found that STAT3 activation was present in approximately fifty percent of Ewing sarcoma tissues as assessed by immunostaining [16]. More recent work investigating the potential role of STAT3 activation in pediatric sarcomas including osteosarcoma (OSA), rhabdomyosarcoma, and Ewing sarcoma demonstrated that constitutive STAT3 phosphorylation occurs in a high percentage of these tumors [1]. Moreover, STAT3 inhibition via a novel small molecule STAT3 inhibitor (STA-21) or a dominant negative form of STAT3 resulted in inhibition of proliferation and apoptosis of sarcoma cell lines expressing high levels of phospho-STAT3 [1]. With respect to OSA, approximately 20% of tumors on an OSA tissue microarray were shown to express high levels of phospho-STAT3 suggesting that this dysregulation is not a consequence of adaptation to tissue culture. As such, STAT3 may represent a target for therapeutic intervention in pediatric OSA.

The ability to rapidly advance therapeutics in pediatric OSA is limited by the low incidence of this disease. However, new therapies are critical as approximately 30–40% of affected patients still die of disease, and no substantial improvement in this outcome has occurred in over ten years. To study human OSA, several animal models have been developed including a variety of transgenic and xenograft rodent models [17-19]. While studies employing these models have been informative, they do not truly recapitulate the biology of OSA that occurs spontaneously in vivo.

OSA occurs in dogs with a frequency far greater than humans (over 10,000 new cases per year in the United States), and evidence suggests that canine OSA exhibits a similar biology to its human counterpart including early metastasis and dysregulated expression of ezrin, Met, and Her2/Neu [20-22]. Additionally, recent work has found that canine and pediatric OSA possess overlapping transcriptional profiles, supporting the notion that these diseases are similar at the molecular level. Indeed, canine OSA has been used as a large animal spontaneous model of the human disease to investigate the clinical efficacy of immunotherapeutic approaches and the activity of an IGF1 inhibitor among several others [23-25].

Given the similarities of canine and pediatric OSA, we investigated the potential role of STAT3 in the canine disease. Our data demonstrate that constitutive activation of STAT3 is present in a substantial subset of canine OSA tumors and human and canine cell lines and that downregulation of STAT3 activity through inhibition of upstream Src family kinases using a small molecule inhibitor (SU6656), direct inhibition of STAT3 DNA binding and transcriptional activities using a novel small molecule inhibitor (LLL3), or modulation of STAT3 expression using siRNAs, all resulted in decreased cell proliferation and viability, ultimately inducing caspase-3 mediated apoptosis in treated cells. Furthermore, inhibition of either Src or STAT3 activity downregulated the expression of survivin, VEGF, and MMP2, all known transcriptional targets of STAT3. These data suggest that STAT3 activation contributes to the survival and proliferation of both human and canine OSA, thereby providing a potentially promising target for therapeutic intervention.

## Methods

### Cell Lines and Reagents

Canine OSA cell lines, OSA7, 8, 11 M, 16, 29, and 32 were provided by Dr. Jaime Modiano (University of Minnesota, Minneapolis, MN). The canine D17 OSA cell line and human OSA cell lines U2OS, SJSA and MG63 were purchased from American Type Cell Culture Collection (ATCC). The canine lines and human line SJSA were maintained in RPMI-1640 supplemented with 10% fetal bovine serum, non-essential amino acids, sodium pyruvate, penicillin, streptomycin, L-glutamine, and HEPES (4-(2-hydroxethyl)-1-piperazineethanesulfonic acid) at 35°C, supplemented with 5% CO_2_. The remaining human cell lines were cultured in DMEM with 10% FBS and same supplements as listed for the canine lines. Normal canine osteoblasts (Cell Applications, Inc, San Diego, CA) were cultured in canine osteoblast medium (also from Cell Applications). The Src inhibitor, SU6656 was purchased from EMD Chemicals (San Diego, CA). The novel STAT3 small molecule inhibitor LLL3 (also known as compound 1), an analog of the small molecule STAT3 inhibitor STA-21, was generously provided by P.K. Li (College of Pharmacy, The Ohio State University, Columbus, OH) [1,26,27]. Concentrations of LLL3 used in experiments were determined after consultation with J. Lin. Canine OSA tumor and normal muscle were obtained from patients treated at The Ohio State University College of Veterinary Medicine Teaching Hospital in compliance with established hospital policies regarding sample collection as part of the Biospecimen Repository at the Teaching Hospital. The protocol for sample collection was approved by the OSU IACUC. Fresh tissue samples were immediately flash frozen in liquid nitrogen and stored in The Ohio State University College of Veterinary Medicine Comparative Oncology Biospecimen Repository. These tumors were evaluated by board certified veterinary pathologists at The Ohio State University College of Veterinary Medicine.

### Western Blotting

To determine the effect of HGF stimulation on STAT3 and Src phosphorylation in normal canine osteoblasts or OSA cells, cells were collected, washed once with PBS, and re-suspended in 1 mL PBS with 50 ng mL^-1 ^rhHGF for 15 minutes or left untreated. To determine the effects of culture conditions on Src and STAT3 phosphorylation, 2–5 × 10^6 ^OSA cells were serum starved for 2 hours while in suspension or adherent culture, or left in serum-containing media while in adherent conditions for 2 hours prior to collection. To determine the downstream effects of Src and STAT3 inhibition, 2 × 10^6 ^OSA cells were treated with SU6656, LLL3, or DMSO and collected after 24, 48, or 72 hours or starved for 2 hours and treated for 2 hours with DMSO or SU6656. Cells were collected then resuspended in lysis buffer consisting of 20 mM Tris-HCl pH 8.0, 137 mM NaCl, 10% glycerol, 1% IPEGAL CA-630, 10 mM ethylenediaminetetraacetic acid (EDTA), 1 mg mL^-1 ^aprotinin, 1 mg mL^-1 ^leupeptin, 1 mg mL^-1 ^pepstatin A, 1 mM phenylmethylsulphonyl fluoride, 1 mM sodium orthovanadate, and 10 mM sodium fluoride (all from Sigma, St. Louis, MO) for 1 hour at 4°C[28]. Additionally, protein lysates were prepared from frozen canine normal muscle or OSA tumor samples by pulverizing tissues in liquid nitrogen and resuspending them in this same lysis buffer with proteinase and phosphatase inhibitors. The results of Western blotting for phosphorylated STAT3 for these samples were from two separate blots. Due to limited sample availability, the Western blots from different tumor samples were batched and run on 2 different occasions and all samples could not be subsequently re-run together on one blot. Following protein quantification via the Bradford Assay, 50 to 100 ug protein was separated by SDS-PAGE, and transferred to PVDF membranes. The membranes were then incubated overnight with anti-p-STAT3 (Y705, Cell Signaling Technology, Danvers, MA), anti-p-Src (Y418, Invitrogen, Carlsbad, CA), anti-PARP (BD Biosciences, San Jose, CA), anti-VEGF (Calbiochem, Gibbstown, NJ), or anti-survivin antibody (Novus Biologicals, Littleton, CO). The membranes were incubated with appropriate horseradish peroxidase linked secondary antibodies, washed, and exposed to substrate (SuperSignal West Dura Extended Duration Substrate, Pierce, Rockford, IL). Blots were stripped, washed, and reprobed for β-actin (Santa Cruz Biotechnology, Santa Cruz, CA), total STAT3 (Cell Signaling Technology, Danvers, MA) or total Src (Cell Signaling Technology, Danvers, MA).

### RT-PCR

For the Src family kinase (SFK) RT-PCR, RNA was extracted from untreated canine OSA cells using TRIzol reagent (Invitrogen, Carlsbad, CA) according to the manufacturer's instructions. For determining levels of mRNA for STAT3 downstream transcriptional targets VEGF and MMP2, human and canine OSA cells were treated with either DMSO, LLL3 (40 uM), or SU6656 (10 uM) for 72 hours and RNA extracted from cells in the same manner. To generate cDNA, 2 μg of total RNA and the M-MLV reverse transcriptase kit (Invitrogen, Carlsbad, CA) were used according to the manufacturer's instructions. Next, 1/20 of the resultant cDNA was used for each PCR reaction in a total volume of 25 μl. Primers designed and utilized for canine SFK members were as follows: (forward/reverse sequences) Src (5'-GGCCCTCTATGACTATGAG/GGTGGTGAGGCGGTGGCACAGGC-3'); Fyn (5'-GCGGCCGGAGGCCAAGGACTC/GTCGCTCAGCATCTTTTCGG-3'); Yes (5'-GATGCTTGGGAAATCCCTCG/GCAGCCCGAAGATCTCGGTG-3'); Lyn (5'-GGTAGCCTTGTACCCCTATG/CTTAATAACATCACCATGCACAGGGTC-3'); and GAPDH (5'-ACCACAGTCCATGCCATCAC/TCCACCACCCTGTTGCTGTA-3'). The annealing temperature utilized for Src and Fyn was 62°C and 58°C was used for all other SFK member PCR reactions. Primers designed and utilized for canine STAT3 transcriptional targets were as follows: (forward/reverse sequences) VEGF (5'-GTCCCAGGCTGCGCCTATGG/GTTTAACTCAAGCTGCCTCGCC-3'); MMP2 (5'-GGAGACTCTCACTTTGATGACG/GGTGAAGGGGAAGACACAGGGG-3'); and GAPDH (5'-ACCACAGTCCATGCCATCAC/TCCACCACCCTGTTGCTGTA-3'). Annealing temperatures for these reactions was 55°C. Primers designed and utilized for the human STAT3 transcriptional target VEGF and GAPDH were as follows: (forward/reverse sequences) VEGF (5'-CCTGGTGGACATCTTCCAGGAGTACCC/CTAATGCCCTGGAGCCTCCC-3') and GAPDH (5'-ACCACAGTCCATGCCATCAC/TCCACCACCCTGTTGCTGTA-3'). An annealing temperature of 60°C was used for all PCR reactions with human primers. All PCR products were run on a 2% agarose gel with ethidium bromide and visualized using the Alpha Imager system (Alpha Innotech Corp, San Leandro, CA).

### STAT3 siRNA transfection

Canine STAT3 small interfering RNA (siRNA) was designed and produced using the Silencer siRNA Construction kit (Ambion, Austin, TX) according to the manufacturer's protocol. Sequences of template canine DNA were as follows: Sense STAT3 siRNA: 5'-AACTCTCTGGTCGACAGTACTCCTGTCTC-3'; Antisense STAT3 siRNA: 5'-AAAGTACTGTCGACCAGAGAGCCTGTCTC-3'; Sense negative control scrambled siRNA: 5'-AAACGTGACACGTTCGGAGAACCTGTCTC-3'; Antisense negative control scrambled siRNA: 5'-AATTCTCCGAACGTGTCACGTCCTGTCTC-3'. 7.5 × 10^4 ^OSA cells were plated and left overnight in 1% serum-containing media. The following day, the media was changed to Opti-MEM media (Invitrogen, Carlsbad, CA) and either media alone, STAT3 or negative control scrambled siRNA (50 pMol of each siRNA) with transfection agent Lipofectamine 2000 (Invitrogen, Carlsbad, CA), or Lipofectamine 2000 alone was added to cells according to manufacturer's protocol. Transfection was repeated again at 48 hours. Cells were collected and processed for Western blotting as described above to detect levels of STAT3, VEGF, survivin, and beta-actin 48 hours after initial transfection. Cell viability was determined at 0, 72, and 96 hours after initial transfection using WST-1 reagent according to the manufacturer's instructions after plating OSA cells as described above (Clontech, Mountain View, CO). Cell viability was calculated as a percentage of the control wells. Additionally, levels of apoptosis were determined at 48 hours using the SensoLyte^® ^Homogeneous AMC Caspase- 3/7 Assay kit (Anaspec Inc, San Jose, CA) as described below after harvesting cell lysate. Images of cells 72 hours post transfection were recorded using digital photography.

### Detection of Apoptosis/Caspase 3,7 Activity

1.1 × 10^4 ^OSA cells were seeded in 96-well plates overnight and incubated with media, DMSO, or increasing concentrations of LLL3 or SU6656 for 24 hours. Wells with media only were included as controls. Levels of caspase- 3/7 activity were determined using the SensoLyte^® ^Homogeneous AMC Caspase- 3/7 Assay kit (Anaspec Inc, San Jose, CA) according to manufacturer's instructions. Briefly, caspase 3/7 substrate solution was added to all wells and incubated prior to measuring fluorescence on a SpectraMax microplate reader (Molecular Devices Corp, Sunnyvale, CA) using excitation at 354 nm and emission detection at 442 nm. Levels of caspase 3/7 activity were reported after subtraction of fluorescence levels of wells with media only.

### EMSA

To confirm that SU6656 and LLL3 impair STAT3 DNA binding, we used the Pierce LightShift Chemiluminescent EMSA kit (Thermo Fisher Scientific Inc, Rockford, IL) that employs a chemiluminescent detection system to detect protein:DNA interactions according to manufacturer's instructions. Briefly, nuclear protein from human and canine OSA cell lines treated 72 hours with media, DMSO, SU6656 10 uM, or LLL3 40 uM was collected using the NucBuster™ Protein Extraction kit (EMD Chemicals Inc, Gibbstown, NJ). Binding reactions using equal amounts of nuclear protein from each treatment group were incubated for 20 minutes at room temperature with DNA probes. The canine STAT3 DNA binding sequence in the promoter for survivin (sense 5'-GCCTTGCATTCCCAGAAGGCCGCGG-3') was used for binding reactions for canine OSA; the STAT3 binding sequence in the human survivin promoter (sense 5'-GAGACTCAGTTTCAAATAAATAAATAAAC-3') was used for the human OSA cell line. Both were purchased from Operon Biotechnologies (Huntsville, AL) in a biotinylated form. Reactions with biotinylated target DNA only and nuclear protein with biotinylated target DNA and excess unlabelled target DNA to compete for binding were included. STAT3 specificity was confirmed by incubation with 6 ug of anti-STAT3 antibody (Santa Cruz Biotechnology Inc, Santa Cruz, CA) to super-shift the protein-DNA complex. Following gel electrophoresis, DNA was transferred to a nylon membrane. The DNA was cross-linked and the biotin-labeled DNA detected by chemiluminescence according to manufacturer's instructions.

### Cell proliferation

2 × 10^3 ^cells were seeded in 96-well plates overnight and incubated with DMSO or increasing concentrations of LLL3 or SU6656 with daily treatment. Each drug concentration was performed in four replicate wells. Media was removed at days 1, 3, and 5 and plates frozen at -80°C overnight before processing with the CyQUANT^® ^Cell Proliferation Assay Kit (Molecular Probes, Eugene, OR) according to manufacturer's instructions. Fluorescence was measured with a SpectraMax microplate reader (Molecular Devices Corp, Sunnyvale, CA) with excitation at 485 nm and emission detection at 530 nm. Cell proliferation was calculated as a percentage of the control (DMSO treated) wells.

### Gel zymography

7.5 × 10^4 ^OSA cells were seeded in 6-well plates overnight and incubated with media only, DMSO, or increasing concentrations of SU6656 or LLL3 for 72 hours. Media was collected and processed and gel zymography performed as described previously [29]. Images were recorded using the Alpha Imager system (Alpha Innotech Corp, San Leandro, CA) and analyzed using Image J.

### Statistical Analysis

All experiments were performed two to three times. Statistical analysis of the data was performed using the Student's t-test. P values of ≤ 0.05 were considered statistically significant.

## Results

### STAT3 is constitutively phosphorylated in OSA tumor tissues and cell lines

**Figure 1:**
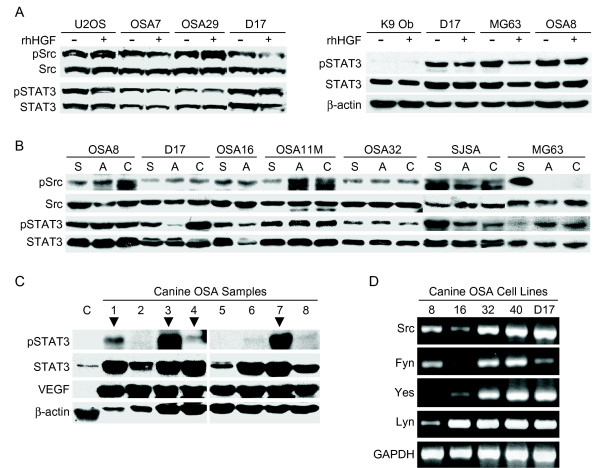
**Activation of Src and STAT3 in OSA cell lines and tissues**. **A) **OSA cells and normal canine osteoblasts were serum starved then left untreated or stimulated with rhHGF (50 ng/ml). Protein lysates were generated and separated by SDS PAGE and Western blotting for pSTAT3 (Y705), pSrc (Y416), total Src, and total STAT3 was performed. **B) **OSA cell lines were serum starved for 2 hours while in suspension culture (S), serum starved for 2 hours while remaining adherent to tissue culture flasks (A), or left in 1% serum while adherent in flasks for 2 hours prior to collection (C). Protein lysates were generated and separated by SDS-PAGE and Western blotting for pSTAT3 (Y705), pSrc (Y416), total Src, and total STAT3 was performed. **C) **Fresh frozen canine OSA tumor tissues and control normal muscle tissue were processed for protein lysates. Protein was separated by SDS-PAGE and Western blotting for pSTAT3 (Y705), STAT3, VEGF, and β-actin was performed. **D) **RNA was collected from canine OSA cell lines and RT-PCR was performed for SFK members Src, Fyn, Yes, and Lyn as well as GAPDH as a control.

STAT3 is known to be activated by various upstream receptor and nonreceptor tyrosine kinases including the Src family kinases (SFKs) and Met [12,30]. We previously generated data suggesting that STAT3 and Src were constitutively phosphorylated in a subset of canine OSA cell lines (OSA8 and D17) and that this phosphorylation was independent of Met signaling. To determine whether activation of the Src-STAT3 pathway is common in OSA cell lines, we first evaluated OSA lines for evidence of Src and STAT3 phosphorylation in the presence or absence of HGF stimulation. As shown in Fig. 1A, both canine OSA lines and the human OSA line U2OS exhibited constitutive Src and STAT3 phosphorylation that was independent of HGF stimulation. We next evaluated normal canine osteoblasts for evidence of STAT3 phosphorylation. In contrast to the malignant OSA lines, normal osteoblasts did not exhibit STAT3 phosphorylation either before or after HGF stimulation (Fig. 1A). To assess whether culture conditions affected the status of STAT3 and Src, cells were grown in suspension or adherent cultures and in the presence or absence of serum and again evaluated for protein phosphorylation. The basal levels of p-STAT3 remained unchanged despite variation in culture conditions in nearly all human and canine OSA cell lines evaluated (Fig. 1B). Basal levels of p-Src were similarly unchanged in most of human and canine OSA cell lines. Additionally, Western blotting of protein lysates derived from fresh frozen canine OSA tumor samples revealed STAT3 phosphorylation in 3/8 tumors (38%) as compared to normal canine muscle (Fig. 1C). Interestingly, levels of total STAT3 expression also varied among tumor samples. This may have been due, in part, to variations in baseline necrosis within the tumor specimens that influenced the proportion of tumor cells to stroma/inflammatory cells. As expected, VEGF was found to be present in all tumor specimens analyzed (Fig. 1C). To begin to assess which Src family members may be responsible for the observed basal Src phosphorylation, we analyzed the expression of Src Family Kinases (SFKs) in 5 canine OSA cell lines by RT-PCR. Although there was some variation in expression levels among the lines, Src, Fyn, Yes, and Lyn were commonly present (Fig 1D).

### STAT3 siRNA induces downregulation of STAT3 and its downstream targets survivin and VEGF with subsequent loss of viability and apoptosis of canine OSA cells

**Figure 2:**
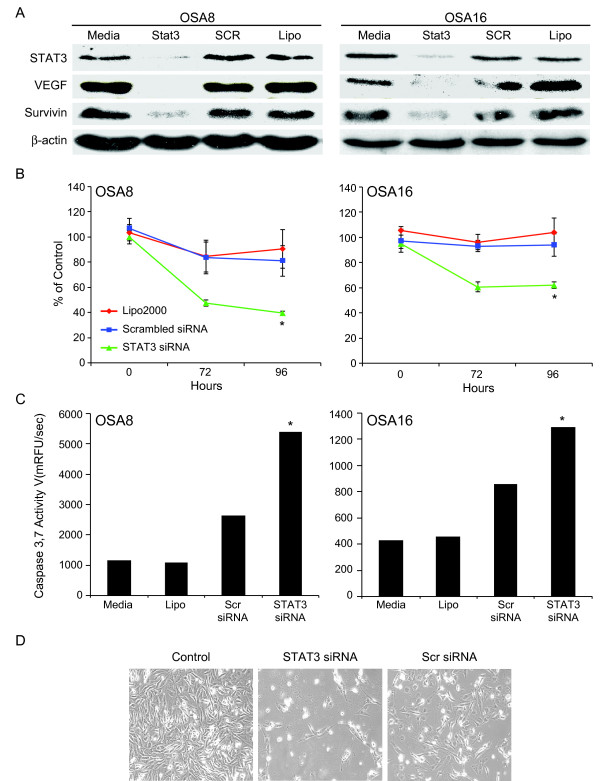
**STAT3 siRNA induces downregulation of STAT3 and its downstream targets with subsequent loss of viability and apoptosis of canine OSA cells**. **A) **Canine OSA cell lines OSA8 and OSA16 were transfected with Lipofectamine 2000 alone, 50 pMol scrambled siRNA, or 50 pMol STAT3 siRNA and collected 48 hours post transfection. Protein lysates were generated and separated via SDS-PAGE. Western blotting for STAT3, VEGF, survivin and β-actin was performed. **B) **OSA8 and OSA16 were transfected with Lipofectamine 2000, scrambled siRNA, or STAT3 siRNA at 0 and 48 hours. Cell viability was assessed at 0, 72, or 96 hours post transfection using the Wst-1 assay. Values are reported as percentage of control wells. **C) **OSA8 and OSA16 were transfected with Lipofectamine 2000, scrambled siRNA, or STAT3 siRNA and generation of active caspase-3/7 was assessed 48 hours post transfection using the SensoLyte^® ^Homogeneous AMC Caspase-3/7 Assay kit. **D) **OSA8 cells were left untreated or transfected with STAT3 siRNA or scrambled siRNA and evaluated by digital photography 72 hours post transfection. *p < 0.05

We designed a small interfering RNA specifically for canine STAT3 to determine the effect of STAT3 downregulation in canine OSA cells. Expression of STAT3 48 hours post transfection was significantly reduced in cells treated with 50 pMol siRNA (Fig. 2A) as evidenced by Western blotting. Additionally, expression of the downstream targets of STAT3, VEGF and survivin, was reduced in both OSA cell lines tested. Downregulation of STAT3 and survivin correlated with a significant loss in viability after 72 and 96 hours and induction of caspase-3/7 activity after 48 hours in both cell lines treated with STAT3 siRNA when compared to those treated with transfection reagent Lipofectamine 2000 alone or scrambled control siRNA (Fig. 2B and 2C). Furthermore, a marked reduction in cellularity occurred at 72 hours post transfection with Stat3 siRNA when compared to cells receiving media alone or scrambled siRNA (Fig. 2D).

### SU6656 inhibits phosphorylation of Src and STAT3 in OSA lines

**Figure 3:**
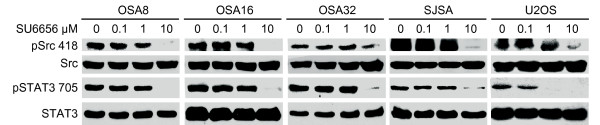
**SU6656 inhibits phosphorylation of Src and STAT3 in OSA lines**. Canine and human OSA cell lines were serum starved then left untreated or incubated with SU6656 for 2 hours. Cells were collected and protein separated by SDS-PAGE, followed by Western blotting for pSrc (Y416), pSTAT3 (Y705), total Src, and total STAT3.

We next evaluated whether the small molecule Src inhibitor SU6656 could inhibit phosphorylation of Src and ultimately that of STAT3 in human and canine OSA cell lines. As shown in Fig. 3, SU6656 downregulated phosphorylation of Src in all canine and human OSA cell lines tested. Additionally, there was an associated downregulation of STAT3 phosphorylation in all human and canine OSA cell lines that corresponded to the loss of pSrc.

### Downregulation of Src or STAT3 leads to decreased STAT3 DNA binding

**Figure 4:**
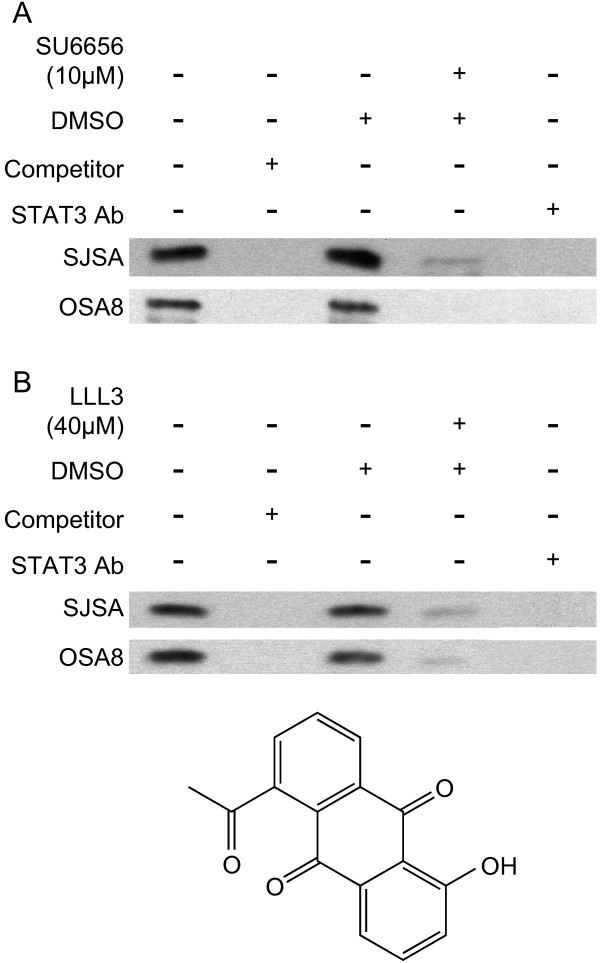
**Downregulation of Src or STAT3 leads to decreased STAT3 DNA binding**. The human OSA cell line SJSA and canine OSA cell line OSA8 were incubated with media, DMSO, or drug (**A) **SU6656, **B) **LLL3) for 72 hours. Cells were harvested and nuclear protein isolated. Nuclear protein was added to binding reactions with labeled species specific DNA probes for the STAT3 recognition sequences located in the promoter for survivin in the presence or absence of unlabelled competitor probe. Additionally, anti-STAT3 antibody was added to nuclear protein from cells treated with media alone to demonstrate specificity of the binding reaction. Reactions were separated on an acrylamide gel, transferred to a nylon membrane, and the DNA was crosslinked. The membranes were processed using the LightShift Chemiluminescent EMSA kit.

STAT3 is known to regulate the expression of many downstream targets important in tumor growth and survival such as survivin and VEGF through its binding to specific sequences in promoter regions of these target genes. To determine whether inhibition of STAT3 activity blocks STAT3 DNA binding activity in OSA cells, we used 2 different approaches. We first interfered with activation by upstream Src using SU6656, as this compound induced a concomitant decrease in pSTAT3 in OSA cells following treatment. As shown in Fig. 4A, there was a dramatic loss of binding to the survivin promoter STAT3 binding sequence in both the canine OSA8 line and human SJSA line after exposure to SU6656 compared to cells treated with DMSO alone. We next directly blocked STAT3 DNA binding and transcriptional activities using a novel small molecule inhibitor of STAT3, LLL3, derived from the previously characterized STA-21 (Fig. 4B) [1,26,27]. LLL3 binds to the side pocket of STAT3 in close proximity to the pTyr705 binding site of the STAT3 monomer, but does not directly bind to pTyr705 which is critical for dimerization [26]. It inhibits STAT3-specific DNA binding activity and STAT3 transcriptional activity (personal communication, J. Lin). As expected, there was a significant loss of STAT3 binding to the STAT3 binding sequence found in the survivin promoter in the OSA8 and SJSA lines following incubation with LLL3 when compared to cells treated with media or DMSO (Fig. 4B). This gel shift was lost when either excess unlabelled competitor DNA probe or STAT3 antibody was added to the binding reactions thus verifying their specificity for STAT3.

### Inhibition of Src or STAT3 decreases cell proliferation, induces caspase-3,7 dependent apoptosis, and downregulates survivin in OSA cell lines

**Figure 5:**
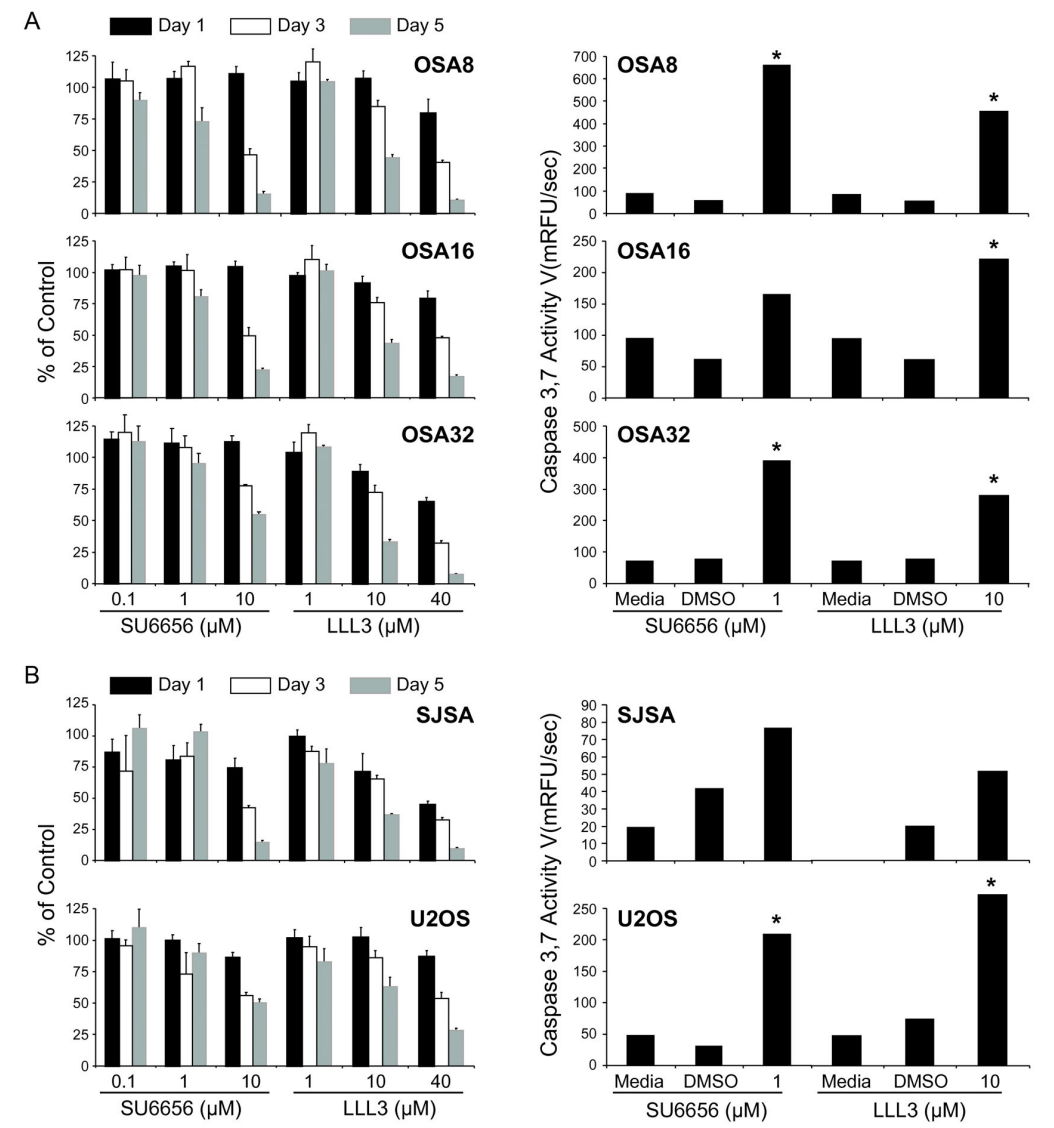
**Inhibition of Src or STAT3 leads to loss of OSA cell proliferation and apoptosis via the caspase 3,7 pathway**. **A) **Canine or **B) **human OSA cell lines were treated with DMSO, SU6656, or LLL3 for 1, 3, or 5 days. Proliferation was analyzed using the CyQUANT cell proliferation assay kit. Apoptosis was assessed by measuring active caspase-3/7 using the SensoLyte^® ^Homogeneous AMC Caspase-3/7 Assay kit. Proliferation values are listed as a percentage of DMSO control. *p < 0.05

**Figure 6:**
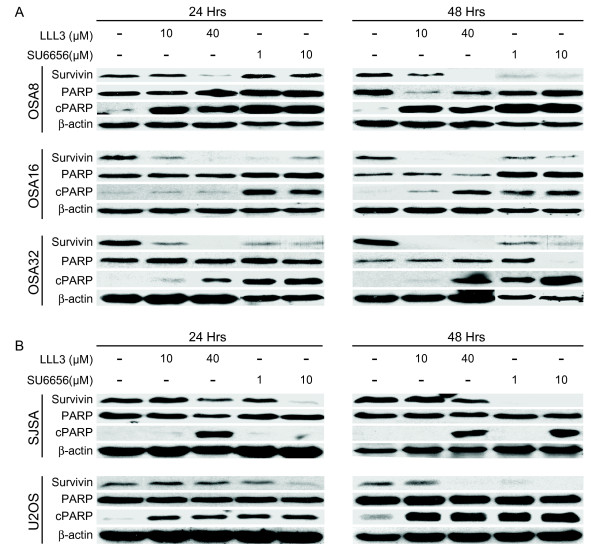
**Inhibition of Src or STAT3 leads to PARP cleavage and downregulates survivin expression in OSA cell lines**. **A) **Canine or **B) **human OSA cell lines were treated for 24 or 48 hours with DMSO, SU6656, or LLL3. Cells were collected and protein lysates were separated via SDS-PAGE. Western blotting for survivin, PARP and β-actin was performed. The anti-PARP antibody utilized in our laboratory has been applied to experiments with canine cell lines and recognizes a 113 kDa intact PARP protein and a 23 kDa cleaved PARP fragment [53].

Given the correlation between dysregulated STAT3 activation and tumor cell survival, we wanted to determine whether inhibition of Src and STAT3 activity in OSA cell lines affected their capacity to proliferate and survive. Human and canine OSA cell lines were cultured with SU6656 or LLL3 at increasing concentrations for 1, 3, or 5 days. Cell proliferation was assessed using the CyQUANT assay and apoptosis was determined using the SensoLyte Homogeneous AMC Caspase 3/7 Assay kit. Although there was some variation in response among cell lines, SU6656 and LLL3 caused a dose and time dependent loss in cell proliferation in all human and canine OSA cell lines (Fig. 5A and 5B). Significant increases in apoptosis as evidenced by caspase 3,7 activity were observed in many OSA cell lines even at 24 hours post treatment (Fig. 5A and 5B). The induction of apoptosis was further confirmed by demonstration of PARP cleavage in OSA cell lines treated with LLL3 or SU6656 for 24 and 48 hours (Fig. 6A and 6B). At both time points, there was a dose and time dependent corresponding decrease in survivin expression in the canine (Fig. 6A) and human OSA cell lines (Fig. 6B).

### Inhibition of Src or STAT3 leads to loss of MMP2 and VEGF expression in OSA cells

**Figure 7:**
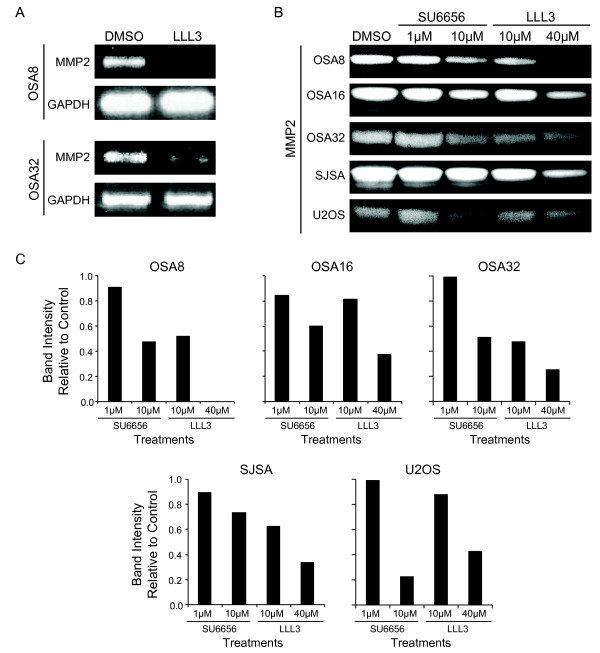
**Downregulation of Src or STAT3 leads to loss of MMP2 expression in OSA cells**. **A) **Canine OSA cell lines OSA8 and OSA 32 were treated with DMSO or LLL3 (40 uM), for 72 hours. RNA was collected and RT-PCR was performed for MMP2 and GAPDH. **B) **Canine and human OSA cell lines were treated with DMSO, SU6656, or LLL3 for 72 hours. Media was collected and MMP2 was assessed via gel zymography. **C) **Gel zymography images were evaluated by densitometry using NIH Image J.

STAT3 is known to regulate the transcription of MMP2, a matrix metalloproteinase known to be important in tumor cell migration and metastasis. Treatment of the canine OSA8 and OSA32 cell lines with the STAT3 inhibitor LLL3 resulted in a significant reduction in MMP2 mRNA expression (Fig. 7A). Treatment of canine and human OSA cell lines with SU6656 or LLL3 for 72 hours resulted in a corresponding dose and time dependent downregulation of both the proenzyme and active form of MMP2 as assessed by gel zymography (Fig. 7B). Changes in MMP2 band intensity following drug treatment were quantified via densitometry using NIH Image J (Fig. 7C).

**Figure 8:**
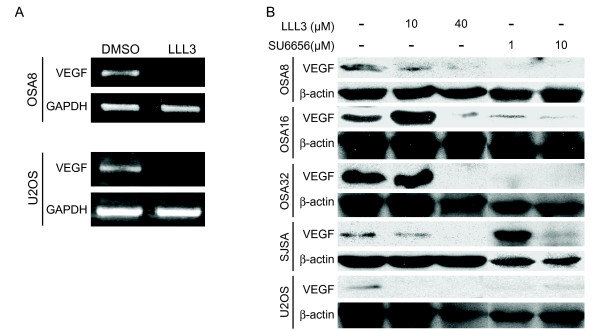
**VEGF expression is reduced after Src or STAT3 inhibition**. **A) **Canine OSA cell line OSA8 and human OSA cell line U2OS were treated with LLL3 for 72 hours. RNA was collected and RT-PCR was performed for VEGF and GAPDH. **B) **Canine and human OSA cell lines were treated with DMSO, SU6656, or LLL3 for 72 hours. Cells were collected and protein lysates were separated via SDS-PAGE. Western blotting for VEGF and β-actin was performed.

Expression of VEGF, another transcriptional target of STAT3 was analyzed after treatment with either the Src inhibitor SU6656 or STAT3 inhibitor LLL3. As with MMP2, treatment of OSA cell lines with LLL3 resulted in a significant reduction in VEGF mRNA expression (Fig. 8A). This translated into a corresponding reduction of VEGF protein expression in canine and human OSA cell lines following exposure to either LLL3 or SU6656 for 72 hrs (Fig. 8B). These results demonstrate that inhibition of STAT3 activity in OSA cells induces not only a direct biologic effect on cell survival, but influences the expression of key proteins critical in metastasis.

## Discussion

The contribution of STAT3 to cancer has been widely confirmed by numerous studies demonstrating dysregulated activation in a variety of human tumors such as breast, lung, pancreatic, ovarian, skin, and renal carcinomas, as well as many hematopoietic tumors [3-5]. Given the prevalence of STAT3 dysregulation in cancer, its role in promoting cell cycle progression, cellular transformation, and prevention of apoptosis, and its unique position downstream of multiple activating tyrosine kinases, STAT3 represents a potential target for therapeutic intervention [31]. While the function of STAT3 in carcinomas and hematopoietic neoplasia has been intensively studied, little is known about the potential role of STAT3 dysregulation in sarcomas. The most detailed work done to date investigating the role of STAT3 in human sarcomas revealed elevated levels of pSTAT3 in a subset of pediatric sarcomas which included osteosarcomas (OSAs) [1] as well as in OSA cancer stem cells [32]. The purpose of this study was to investigate the role of STAT3 dysregulation in OSA and to explore the biologic consequences of STAT3 inhibition in OSA cell lines.

Several animal models have been developed to study human OSA including radiation-induced OSA in Sprague-Dawley rats [19], subcutaneous [18] and orthotopic [17] implantation of OSA cells in mice, as well as a variety of transgenic mouse models that develop OSA spontaneously. While studies employing these models have been informative, they do not truly recapitulate the biology of OSA that occurs spontaneously *in vivo*, particularly with respect to the development of appendicular OSA and chemotherapy-resistant metastases.

While OSA is the most common malignant bone tumor in children, it occurs with a far greater frequency in the canine population (1000 cases per year compared to greater than 10,000 cases per year in the United States). Beyond clinical similarities such as tumor location and chemotherapy resistance, canine and human OSA share similar biologic features including early metastasis, dysregulated expression of ezrin, Met, and Her2/Neu, and overlapping transcriptional profiles [20-22,28,33-35].

Spontaneous canine OSA has been used to evaluate novel therapeutics such as liposome encapsulated muramyl tripeptide, IGF-1R inhibitors, and more recently, rapamycin. [23,36-39]. Together, these data support the idea that canine OSA is a relevant, spontaneous, large animal model of human OSA that closely recapitulates the biology of human OSA. As such, we chose to evaluate both human and canine OSA cell lines, as well as fresh canine OSA tumors for evidence of STAT3 dysregulation as demonstration of abnormalities in both species would lay the foundation for preclinical testing of STAT3 inhibitors in dogs with OSA.

Constitutive phosphorylation of Src and STAT3 was detected by Western blotting in all human and canine OSA cell lines tested despite alterations in culture conditions as well as in a subset (38%) of fresh frozen OSA derived from canine patients, but not in normal canine osteoblasts. This finding supports previous studies in which human OSA cell lines and 19% of tissue microarray samples were positive for elevated STAT3 phosphorylation [1]. Indeed, all human and canine OSA cell lines tested expressed survivin, MMP2 and VEGF, downstream targets of STAT3 important in preventing apoptosis, enhancing invasion, and promoting metastasis. Increased expression of survivin has been associated with reduced survival in OSA [40] as well as chemotherapy and radiotherapy resistance. Expression of VEGF in OSA is also strongly predictive for pulmonary metastasis and poor prognosis [41]. These data support the notion that constitutive activation of both Src and STAT3 is common in both canine and human OSA.

Studies have shown that inhibition of STAT3 activation through various methods such as RNA interference, double-stranded decoy oligodeoxynucleotides, and small molecule inhibitors results in decreased viability and apoptosis of a variety of human tumor cell lines, including those derived from sarcomas [1,42-46]. Previous work has shown that downregulation of STAT3 using a dominant-negative form decreased viability of human OSA cells resulting in induction of apoptosis [1]. Additionally, STAT3 knockdown using RNA interference in colon cancer cell lines reduced survivin expression resulting in anoikis [47]. Transfection of canine OSA cell lines with STAT3 siRNA led to loss of STAT3, survivin, and VEGF expression with subsequent decreases in cell proliferation and induction of apoptosis through the caspase 3/7 pathway. Similar effects were seen in both canine and human OSA cell lines treated with the small molecule Src inhibitor SU6656 and the novel small molecule STAT3 inhibitor LLL3. These data are consistent with findings of previous studies of STAT3 inhibition in human carcinoma and leukemia cell lines.

Abrogation of STAT3 DNA binding in both human and canine OSA cell lines after treatment with both inhibitors, as shown by EMSA, delineates the mechanism of loss of downstream target mRNA and protein expression. Interestingly, the STAT3 small molecule inhibitor STA-21, the compound from which LLL3 was derived, was found to act in a similar manner, preventing STAT3 activity by blocking dimerization, translocation into the nucleus, and execution of its signaling pathway in breast cancer cells at roughly the same concentration [27]. Treatment with STA-21 suppressed cell growth, reduced cell number, and induced apoptosis in human sarcomas through caspase 3, 8, and 9 dependent pathways [1]. These results support the notion that the biologic effects manifested in OSA cell lines following either loss of total STAT3 or loss of STAT3 DNA binding are directly related to downregulation of various STAT3 transcriptional targets responsible for survival.

To correlate the effects of Src family kinase inhibition with STAT3 activation, we evaluated the effects of inhibition of Src on OSA cell lines. Previous work has shown that the small molecule SFK inhibitor SU6656 blocks the phosphorylation of Src, Fyn, Lyn, and Yes with approximately equal potencies [48]. SU6656 has been shown to decrease activation of STAT3 and subsequent downstream targets in NIH3T3 cells resulting in cell cycle arrest and apoptosis in human lung cancer cells [30,49]. Our data demonstrated that not only are these SFK members expressed in the canine OSA cell lines tested, but that inhibition of Src phosphorylation in both human and canine OSA cell lines with SU6656 abrogated Src signaling resulting in a concomitant decrease in STAT3 phosphorylation. These data suggest that Src contributes to activation of STAT3 in the human and canine OSA cell lines and that inhibition of Src represents another method for abrogating STAT3 activity in OSA. Indeed, small molecule SFK inhibitors have been evaluated in human OSA and Ewing's sarcoma resulting in suppression of tumor cell migration, apoptosis of tumor cell lines, and inhibition of tumor growth in xenograft models [46,47,50-52]. Given the apparent role of Src in STAT3 activation, it is possible that SFK inhibitors are working, in part, through modulation of STAT3 phosphorylation.

## Conclusion

Our data demonstrate that constitutive activation of STAT3 is present in both canine and human OSA cell lines and in a subset of canine OSA fresh tumor specimens. Downregulation of STAT3 activity in OSA cell lines, either directly or through Src inhibition reduced proliferation and expression of STAT3 transcriptional targets, and induced apoptosis through caspase-3/7 activation. Data generated in both the canine and human OSA cell lines were concordant, suggesting that dysregulation of STAT3 may be common in this disease. This work serves as the foundation for future experiments with LLL3 and other inhibitors of STAT3 both in mouse models of OSA with eventual application in dogs with spontaneous OSA, as a prelude to clinical work in children.

## Competing interests

The authors declare that they have no competing interests.

## Authors' contributions

SF designed and carried out molecular experiments on OSA tissues and cell lines and drafted the manuscript. AL and JM conducted immunoblotting experiments on OSA cell lines. MB participated in RT-PCR design and performance. JL and PL provided LLL3 and assisted in design of experiments. WK provided OSA tumor tissues and assisted in experimental design. CL conceived of the study, assisted in experimental design, and helped draft the manuscript. All authors read and approved the final manuscript.

## Pre-publication history

The pre-publication history for this paper can be accessed here:

http://www.biomedcentral.com/1471-2407/9/81/prepub
